# Classification of root canal microorganisms using electronic-nose and discriminant analysis

**DOI:** 10.1186/1475-925X-9-77

**Published:** 2010-11-22

**Authors:** Bekir H Aksebzeci, Musa H Asyalı, Yasemin Kahraman, Özgür Er, Esma Kaya, Hatice Özbilge, Sadık Kara

**Affiliations:** 1 Erciyes University, Faculty of Engineering, Department of Electrical & Electronics Eng., 38039 Kayseri, Turkey; 2 Zirve University, Faculty of Engineering, Department of Electrical & Electronics Eng., 27260 Gaziantep, Turkey; 3 Erciyes University, Faculty of Dentistry, Department of Conservative Dentistry and Endodontics, 38039 Kayseri, Turkey; 4 Erciyes University, Faculty of Pharmacy, Department of Microbiology, 38039 Kayseri, Turkey; 5 Fatih University, Faculty of Engineering, Department of Electrical & Electronics Eng., 34500 Istanbul, Turkey

## Abstract

**Background:**

Root canal treatment is a debridement process which disrupts and removes entire microorganisms from the root canal system. Identification of microorganisms may help clinicians decide on treatment alternatives such as using different irrigants, intracanal medicaments and antibiotics. However, the difficulty in cultivation and the complexity in isolation of predominant anaerobic microorganisms make clinicians resort to empirical medical treatments. For this reason, identification of microorganisms is not a routinely used procedure in root canal treatment. In this study, we aimed at classifying 7 different standard microorganism strains which are frequently seen in root canal infections, using odor data collected using an electronic nose instrument.

**Method:**

Our microorganism odor data set consisted of 5 repeated samples from 7 different classes at 4 concentration levels. For each concentration, 35 samples were classified using 3 different discriminant analysis methods. In order to determine an optimal setting for using electronic-nose in such an application, we have tried 3 different approaches in evaluating sensor responses. Moreover, we have used 3 different sensor baseline values in normalizing sensor responses. Since the number of sensors is relatively large compared to sample size, we have also investigated the influence of two different dimension reduction methods on classification performance.

**Results:**

We have found that quadratic type dicriminant analysis outperforms other varieties of this method. We have also observed that classification performance decreases as the concentration decreases. Among different baseline values used for pre-processing the sensor responses, the model where the minimum values of sensor readings in the sample were accepted as the baseline yields better classification performance. Corresponding to this optimal choice of baseline value, we have noted that among different sensor response model and feature reduction method combinations, the difference model with standard deviation based dimension reduction or normalized fractional difference model with principal component analysis based dimension reduction results in the best overall performance across different concentrations.

****Conclusion**:**

Our results reveal that the electronic nose technology is a promising and convenient alternative for classifying microorganisms that cause root canal infections. With our comprehensive approach, we have also determined optimal settings to obtain higher classification performance using this technology and discriminant analysis.

## 1. Introduction

Endodontics is largely concerned with the treatment of infections originating in the root canal system. Microorganisms are the primary etiological agent for the root canal system infections and periapical lesions [1,2]. Dental infections may be successfully treated by the removal of the source of the infection [3]. Since W. D. Miller [4] demonstrated the presence of bacteria in necrotic pulp tissue, the role of the oral microflora in the pathogenesis of pulpal and periapical pathosis has become increasingly evident. Although more than 300 species of bacteria have been isolated from the oral cavity, only a limited number have been consistently isolated from endodontic infections [5]. These include species of the genera *Streptococcus, Fusobacterium, Prevotella, Porphyromonas, Eubacterium, Peptostreptococcus, Bacteroides*, and *Lactobacillus *[5,6]. Several studies have also reported cultivation of fungi from endodontic infections [7,8].

Clinicians must understand the close relationship between the presence of microorganisms and endodontic disease process in order to develop an effective rationale for treatment. Culture and molecular methods can be used to detect bacterial species in root canal infections. Culture method identifies the predominant species of endodontic infections. Cultivation is important in terms of which microorganism the clinician is facing. Molecular techniques have been used to detect microorganism in endodontic infections using DNA-DNA hybridization analysis and polymerase chain reaction method. However, culture and molecular methods are laborious and expensive, they are often slow to provide a diagnostic result [9-13]. Especially, conventional diagnosis of anaerobic bacteria that is generally responsible for root canal infections is difficult, due to requirements of long cultivation time for these bacteria, special equipment and experienced staff. Because of difficulties in cultivation process of anaerobic microorganisms, identification of these microorganisms is not routinely used. Clinicians tend to resort to empiric decision making and/or treatment.

Electronic noses (e-noses) have been used for the analysis of odors or volatile organic compounds. E-noses consist of sensing and pattern recognition systems. Sensing system of an e-nose is formed as an array of chemical gas sensors. Chemical gas sensors contain a chemical detection layer and transform a chemical interaction into an electrical signal [14]. Selectivity of an e-nose is also related to the particular pattern recognition techniques applied to the responses from the chemical gas sensors [15].

E-nose technology has been used in various microbiological studies [16-20]. In a previous study, we have classified 8 different microorganism cultures in petri dishes using e-nose [21]. These microorganisms were aerobic type. In this study, we have focused on specific standard microorganism strains which are frequently encountered in root canal. Many of these microorganisms are anaerobic; so isolation and cultivation processes were relatively difficult. After all microorganism species were grown in their own specific agar, they were transferred into liquid medium. We have then taken odor samples from these microorganism suspensions and formed datasets with respect to various pre-processing and dimension reduction methods. We have obtained successful classification results using the discriminant analysis method which is used extensively in the literature.

Main contributions of this study are two fold. Firstly, we have suggested a new application area for the use of e-nose technology. To be more specific, we have demonstrated that use of a classification method such as discriminant analysis on odor data collected through an e-nose device is a feasible alternative for detection of microorganisms that cause root canal infections. Secondly, with our comprehensive approach, we have determined optimal settings to obtain higher classification performance using e-nose technology and discriminant analysis.

## 2. Materials and Methods

### 2.1. Microorganisms

In this study, we have used 7 different standard microorganism strains (*Candida albicans, Candida glabrata, Fusobacterium nucleatum, Porphyromonas gingivalis, Pseudoramibacter alactolyticus, Streptococcus sanguinis, Enterococcus faecalis*). Microorganism strains we have grown locally (at the Faculty of Pharmacy, Erciyes University, Kayseri) belong to the American Type Culture Collection (ATCC), the German Collection of Microorganisms and Cell Cultures (DSMZ) and the Refik Saydam Culture Collection (RSKK) at Refik Saydam Hygiene Center in Ankara. Names of microorganism species, oxygen requirements, growth medium, incubation conditions and collection types are presented in Table 1. All microorganisms were grown on their specific medium in standard petri dishes at 37°C. After culturing, colonies inoculated to vials different concentrations were adjusted to be at 12 × 10^8 ^cfu/ml (4 McFarland) in 4 ml sterile saline solution using PhoenixSpec Nephelometer (BD Diagnostic Systems, Franklin Lakes, NJ USA) [22]. Microorganism suspensions were prepared and diluted -in sterile glass tubes of standard size- to be of 12 × 10^5^, 12 × 10^3^, and 12 × 10^1 ^cfu/ml concentration.

**Table 1:** Names and descriptions of the microorganism species used in the study.

Microorganism	Species	**O**_**2 **_**Requirement**	Growth Medium	Incubation Condition	Class
*Candida albicans*	Yeast	Aerobe	Sabouraud dextrose agar	24-48 hours at 37°C	ATCC 90028
*Candida glabrata (Torulopsis glabrata)*	Yeast	Aerobe	Sabouraud dextrose agar	24-48 hours at 37°C	RSKK 04019
*Fusobacterium nucleatum*	Bacteria	Anaerobe	Anaerobic blood agar	4-6 days at 37°C	DSMZ 20482
*Porphyromonas gingivalis*	Bacteria	Anaerobe	Anaerobic blood agar	4-6 days at 37°C	ATCC 33277
*Pseudoramibacter alactolyticus*	Bacteria	Anaerobe	Anaerobic blood agar	4-6 days at 37°C	DSMZ 3980
*Streptococcus sanguinis*	Bacteria	Facultative anaerobe	Tryptic soy agar	4 days at 37°C %5 CO_2_	DSMZ 20567
*Enterococcus faecalis*	Bacteria	Facultative anaerobe	Blood agar	24 hours at 37°C	ATCC 29212

### 2.2. Instrumentation

The application of microorganism classification using Cyranose 320 e-nose (Smiths Detection, Hertfordshire, UK) was investigated in this study [23]. Cyranose 320 is a portable e-nose instrument, which has 32 individual carbon-black polymer composite sensors configured into an array. Measurements are based on change in the resistance of each sensor when exposed to an odor. Real-time sensor resistance values in the sensor array are transferred to the PC using a data cable.

### 2.3. Test Procedure

**Figure 1:**
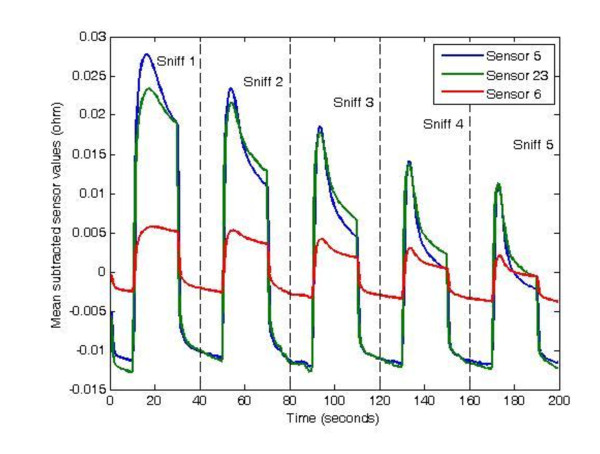
**The data obtained from 5 repeated smells from a sample of *Candida albicans***. (Only three sensor responses that undergo maximum changes are shown.)

We have taken 5 repeated odor samples from each microorganism suspensions at 4 different concentration levels. That is, we have had 35 odor samples at each concentration level. Before collecting odor data with the e-nose, the cap of each vial that contained the suspension was unscrewed and put into a sealed plastic bag. Then the probe of e-nose was inserted into the bag. We waited for 5 minutes to let the sample disperse into the bag for each data collecting process. The accompanying data acquisition software of the e-nose was set in such a way that an odor sample is collected in 40 seconds. Figure 1 shows data of 5 odor samples obtained from *Candida albicans *at 12 × 10^8 ^cfu/ml concentration using Cyranose 320. (Only the data belonging 3 sensors that demonstrated maximum changes are shown.)

### 2.4. Data Pre-processing

An odor sample actually consists of 74 readings that include instantaneous resistance values of 32 sensors on the e-nose. Let *i *= 1, 2, ..., 35 be the sample, *k *= 1, 2, ..., 74 be reading, and *j *= 1, 2, ..., 32 be the sensor index, then each raw odor sensor data R*_i _*= [*r_i, k, j_*] constitute a 74-by 32 matrix.

$$R_{i}=\left[\begin{array}{ccccc} r_{i,1,1} & r_{i,1,2} & ... & ... & r_{i,1,32}\\ r_{i,2,1} & r_{i,2,2} & ... & ... & r_{i,2,32}\\ ... & ... & ... & ... & ...\\ ... & ... & r_{i,k,j} & ... & ...\\ r_{i,74,1} & r_{i,74,2} & ... & ... & r_{i,74,32} \end{array}\right]$$

**Figure 2:**
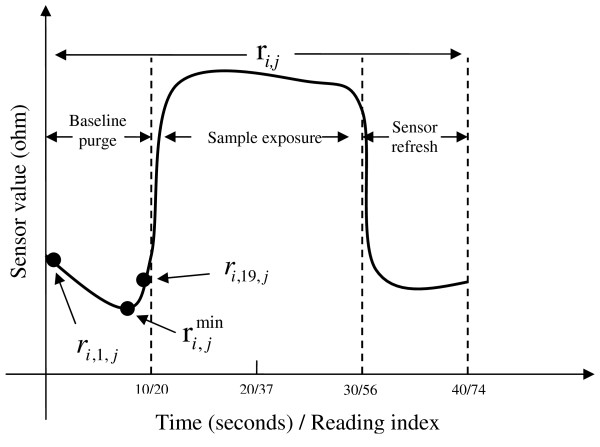
**Illustration of different baseline values and phases of an odor sample**. (Only one sensor response is shown.)

An odor sample acquisition session of 40 seconds consists of 3 phases. In the first 10 seconds, the sensors of e-nose were cleaned with indoor air (baseline purge). The first 19 rows of the sample matrix R*_i _*correspond to this phase. In the following 20 seconds, the odor of microorganism suspension was applied to the sensor chamber (sample exposure). The rows between 20 and 56 of the sample matrix R*_i _*correspond to this phase. The last 10 seconds, the sensor chamber was cleaned again with indoor air (sensors refresh) represents the rest of the data. Figure 2 shows these 3 phases of data acquisition along with some different alternatives for the baseline value which will be used in the pre-processing.

Concerning the resistance baseline values of sensors, 3 different approaches were considered:

• Baseline Value-1: The minimum values of sensor readings in the odor sample.

(1) $${\text{R}}_{i}^{\text{o}}={\text{R}}_{i}^{\min}$$

• Baseline Value-2: The first values of sensor readings in the odor sample (*k *= 1).

(2) $${\text{R}}_{i}^{\text{o}}=[r_{i,1,j}]={\text{R}}_{i}^{\text{first}}$$

• Baseline Value-3: The final values of sensor readings in the baseline purge section of odor sample (*k *= 19).

(3) $${\text{R}}_{i}^{\text{o}}=[r_{i,19,j}]={\text{R}}_{i}^{\text{bp}}$$

**Figure 3:**
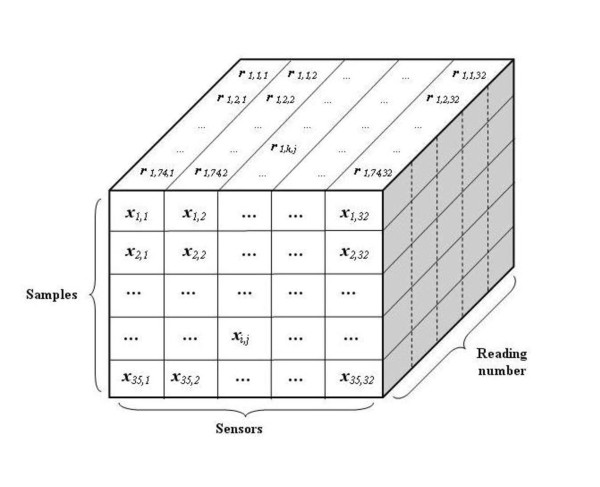
**Data cube representing microorganism odor data**.

We have also used 3 different sensor response models in order to remove the time (i.e., the vertical) dimension in the sample matrix R*_i _*and convert or reduce it into a x*_i _*= [*x_i, j_*], 1-by-32 vector. Thus our microorganism odor dataset will be a X = [*x_i, j_*], 35-by-32 data matrix. Figure 3 shows the data cube representing our odor data.

• Difference model: The difference between maximum (${\text{R}}_{i}^{\text{max}}$ is a row vector containing the maximum element from each column of R*_i_*.) and baseline resistance values (${\text{R}}_{i}^{\text{o}}$) of the sensors.

(4) $${\text{x}}_{i}={\text{R}}_{i}^{\max}-{\text{R}}_{i}^{\text{o}}=\Delta {\text{R}}_{i}$$

• Fractional difference model: The ratio of sensor resistance change to baseline value.

(5) $${\text{x}}_{i}=\Delta {\text{R}}_{i}{\text{ / R}}_{i}^{\text{o}}$$

• Normalized fractional difference model: The normalization of fractional difference model based on the sensor that demonstrates the maximum change.

(6) $${\text{x}}_{i}=(\Delta {\text{R}}_{i}{\text{/R}}_{i}^{\text{o}})/{\left(\Delta {\text{R}}_{i}{\text{/R}}_{i}^{\text{o}}\right)}^{\max}\;$$

Thus total of 9 different data pre-processing methods (combinations) were applied to the odor samples from 7 microorganisms. Equations and abbreviations of these pre-processing methods are given in Table 2.

**Table 2:** Equations and abbreviations of pre-processing methods.

Baseline Value	Sensor Response Model	Equation	Abbreviation
Baseline value-1 ${\text{R}}_{i}^{\text{o}}={\text{R}}_{i}^{\min}$	Difference model	${\text{x}}_{i}={\text{R}}_{i}^{\max}-{\text{R}}_{i}^{\text{min}}=\Delta {\text{R}}_{i}$	d1
	
	Fractional difference model	${\text{x}}_{i}=\Delta {\text{R}}_{i}{\text{ / R}}_{i}^{\min}$	fd1
	
	Normalized fractional difference model	${\text{x}}_{i}=(\Delta {\text{R}}_{i}{\text{/R}}_{i}^{\text{min}})/{\left(\Delta {\text{R}}_{i}{\text{/R}}_{i}^{\text{min}}\right)}^{\max}\;$	nfd1

Baseline value-2 ${\text{R}}_{i}^{\text{o}}={\text{R}}_{i}^{\text{first}}$	Difference model	${\text{x}}_{i}={\text{R}}_{i}^{\max}-{\text{R}}_{i}^{\text{first}}=\Delta {\text{R}}_{i}$	d2
	
	Fractional difference model	${\text{x}}_{i}=\Delta {\text{R}}_{i}{\text{ / R}}_{i}^{\text{first}}$	fd2
	
	Normalized fractional difference model	${\text{x}}_{i}=(\Delta {\text{R}}_{i}{\text{/R}}_{i}^{\text{first}})/{\left(\Delta {\text{R}}_{i}{\text{/R}}_{i}^{\text{first}}\right)}^{\max}\;$	nfd2

Baseline value-3 ${\text{R}}_{i}^{\text{o}}={\text{R}}_{i}^{\text{bp}}$	Difference model	${\text{x}}_{i}={\text{R}}_{i}^{\max}-{\text{R}}_{i}^{\text{bp}}=\Delta {\text{R}}_{i}$	d3
	
	Fractional difference model	${\text{x}}_{i}=\Delta {\text{R}}_{i}{\text{ / R}}_{i}^{\text{bp}}$	fd3
	
	Normalized fractional difference model	${\text{x}}_{i}=(\Delta {\text{R}}_{i}{\text{/R}}_{i}^{\text{bp}})/{\left(\Delta {\text{R}}_{i}{\text{/R}}_{i}^{\text{bp}}\right)}^{\max}\;$	nfd3

As the number of samples is relatively small compared to the feature size (35 versus 32), in order to avoid the curse of dimensionality problem and have an efficient classification setting, we have employed 2 different dimension reduction methods to have only 3 features. These methods were as follows:

• Choose 3 sensors that demonstrate the maximum change on the basis of standard deviation (std),

• Choose the first 3 principal components following the application of Principal Components Analysis (PCA).

As a result, 18 different datasets were obtained by applying different pre-processing and dimension reduction methods to the raw odor data. Finally, 3 different Discriminant Analysis (DA) methods (linear, Mahalanobis distance and quadratic) were used in order to classify each dataset into 7 groups.

### 2.5. Discriminant Analysis

Given a multi-category classification problem, let **x **denote an observation in a *d*-dimensional feature space with a probability density function *p*(**x**) (evidence factor), *i *= 1, 2, ..., *c *be the class index, and *ω_i _*be the class labels indicating the true state of the nature. In our study *c *is 7, since we have 7 microorganism species to be classified.

If class prior probabilities *P*(*ω_i_*) and the class conditional probability density functions (likelihood) *p*(**x**|*ω_i_*) are known, then the posterior probabilities are calculated using Bayes formula:

(7) $$P(\omega_{i}|\text{x})=\frac{p(\text{x}|\omega_{i})P(\omega_{i})}{p(\text{x})}.$$

Bayes decision rule basically assigns **x **to the class *ω_i _*that has the highest posterior probability:

(8) $$\text{Decide}\;\omega_{i}\;if\;P(\omega_{i}|\text{x})>P(\omega_{j}|\text{x})\;\text{for}\;\text{all}\;j\neq i.$$

In Eq. (7) the evidence *p*(**x**) is just a scaling factor thereby it is unimportant in the case of decision making. For the minimum error rate classification, we can use natural logarithm transform of posterior probabilities. The following equation maximizes the posterior probability so it defines maximum discriminant function:

(9) $$g_{i}(\text{x})=\ln p(\text{x}|\omega_{i})+\ln P(\omega_{i}).$$

If the class conditional densities *p*(**x**|*ω_i_*) are multivariate normal, that is

(10) $$p(\text{x}|\omega_{i})=\frac{1}{{\left(2\pi \right)}^{d/2}|\Sigma_{i}{|}^{1/2}}\exp(-\frac{{\left(\text{x}-\mu_{i}\right)}^{t}\Sigma_{i}^{-1}(\text{x}-\mu_{i})}{2}).$$

where ***μ**_i _*is the *d *× 1 mean vector, **Σ***_i _*is the *d *× *d *covariance matrix, and |**Σ***_i_*| is the determinant of the covariance matrix, then the discriminant function can be written as:

(11) $$g_{i}(\text{x})=-\frac{1}{2}[d\ln(2\pi )+\ln|\Sigma_{i}|+{\left(\text{x}-\mu_{i}\right)}^{t}\Sigma_{i}^{-1}(\text{x}-\mu_{i})]+\ln P(\omega_{i}).$$

We have examined this discriminant function for three special cases [24]. All of these classification algorithms were implemented using Matlab R2007b (Mathworks Inc., Natick, MA) software [25].

#### Case 1: Linear

In this case, it is assumed that each class has the same (pooled) covariance matrix (i.e., **Σ***_i _= ***Σ**). Hence *d*ln (2*π*) and |**Σ***_i_*| terms in Eq. (11) are independent of *i*, so these can be ignored. Then, the discriminant function becomes:

$$g_{i}(\text{x})=-\frac{1}{2}{\left(\text{x}-\mu_{i}\right)}^{t}\Sigma^{-1}(\text{x}-\mu_{i})+\ln P(\omega_{i}).$$

Then, we expand the first term on the right hand side

$${\left(\text{x}-\mu_{i}\right)}^{t}\Sigma^{-1}(\text{x}-\mu_{i})={\text{x}}^{t}\Sigma^{-1}\text{x}-{\text{x}}^{t}\Sigma^{-1}\mu_{i}-\mu_{i}^{t}\Sigma^{-1}\text{x}+\mu_{i}^{t}\Sigma^{-1}\mu_{i}.$$

The quadratic term **x*^t ^*Σ^-1 ^x **is independent of *i*, so it can be ignored too. Further, like other terms in the expansion, **x*^t ^*Σ^-1 ^μ*_i _***is a scalar term and hence ${\left({\text{x}}^{t}\Sigma^{-1}\mu_{i}\right)}^{t}={\text{x}}^{t}\Sigma^{-1}\mu_{i}=\mu_{i}^{t}\Sigma^{-1}\text{x}$. Thus, the discriminant function can be reduced to the following expression which is linear in **x**:

(12) $$g_{i}(\text{x})=\mu_{i}^{t}\Sigma^{-1}\text{x}-\frac{1}{2}\mu_{i}^{t}\Sigma^{-1}\mu_{i}+\ln P(\omega_{i}).$$

#### Case 2: Mahalanobis Distance

In this case, the covariance matrices for all of the classes are different and the discriminant function is simply the Mahalanobis distance part of Eq. (11), that is

(13) $$g_{i}(\text{x})={\left(\text{x}-\mu_{i}\right)}^{t}\Sigma_{i}^{-1}(\text{x}-\mu_{i}).$$

#### Case 3: Quadratic

In this case, the covariance matrices are assumed to be different for different classes, i.e., **Σ***_i _*is arbitrary. Only the *d *ln(2*π*) ln term in Eq. (11) is independent of *i*, so it can be dropped, and the discriminant function is:

(14) $$g_{i}(\text{x})=-\frac{1}{2}{\left(\text{x}-\mu_{i}\right)}^{t}\Sigma_{i}^{-1}(\text{x}-\mu_{i})-\frac{1}{2}\ln|\Sigma_{i}|+\ln P(\omega_{i}).$$

## 3. Results

The odor data (35 samples) in certain concentrations were classified according to the type of microorganism species. Table 3 summarizes our classification results in terms of training error. For each concentration, different pre-processing and dimension reduction methods were applied to the odor data, resulting in 18 different datasets, corresponding to the rows of the table. These datasets were classified into 7 groups using linear, Mahalanobis distance, and quadratic type DA classifiers. When we examine the classification performance of 3 methods, the 'quadratic' method consistently offers the best results across different concentrations, pre-processing and dimension reduction methods. Hence we have made a reduced version of Table 3, namely Table 4, where we have only included the results for the 'quadratic classifier.' However, this time, in order to examine the influence of different factors on the classification performance; we have included averages of classification errors across different dimensions in the table.

**Table 3:** Classification error rates (%) at different concentrations using Linear (L), Mahalanobis (M), and Quadratic (Q) type DA methods.

	Concentration											
	
Dataset (pre-processing & dimension reduction)	12 × 10^8 ^cfu/ml			12 × 10^5 ^cfu/ml			12 × 10^3 ^cfu/ml			12 × 10^1 ^cfu/ml		
	
	L	M	Q	L	M	Q	L	M	Q	L	M	Q
**d1 & std**	14.29	2.86	**0.00**	25.71	5.71	**0.00**	31.43	5.71	2.86	20.00	8.57	2.86

**d1 & pca**	5.71	**0.00**	**0.00**	25.71	5.71	2.86	22.86	5.71	**0.00**	17.14	11.43	5.71

**fd1 & std**	17.14	8.57	2.86	25.71	2.86	5.71	25.71	5.71	2.86	5.71	2.86	**0.00**

**fd1 & pca**	5.71	2.86	**0.00**	17.14	**0.00**	**0.00**	34.29	8.57	8.57	20.00	5.71	**0.00**

**nfd1 & std**	11.43	17.14	**0.00**	**0.00**	**0.00**	**0.00**	22.86	8.57	5.71	8.57	2.86	5.71

**nfd1 & pca**	11.43	17.14	**0.00**	**0.00**	**0.00**	**0.00**	22.86	2.86	2.86	11.43	5.71	2.86

**d2 & std**	34.29	8.57	2.86	17.14	8.57	11.43	22.86	5.71	8.57	25.71	17.14	11.43

**d2 & pca**	31.43	**0.00**	**0.00**	17.14	8.57	11.43	31.43	14.29	8.57	34.29	28.57	22.86

**fd2 & std**	17.14	2.86	**0.00**	17.14	**0.00**	**0.00**	37.14	14.29	17.14	20.00	14.29	14.29

**fd2 & pca**	20.00	2.86	2.86	14.29	5.71	2.86	34.29	2.86	2.86	25.71	8.57	2.86

**nfd2 & std**	14.29	5.71	5.71	8.57	2.86	2.86	22.86	2.86	2.86	5.71	2.86	2.86

**nfd2 & pca**	14.29	8.57	**0.00**	8.57	2.86	**0.00**	14.29	8.57	5.71	14.29	8.57	2.86

**d3 & std**	20.00	5.71	**0.00**	28.57	**0.00**	2.86	28.57	8.57	2.86	25.71	14.29	5.71

**d3 & pca**	8.57	**0.00**	**0.00**	31.43	5.71	**0.00**	25.71	8.57	2.86	17.14	11.43	11.43

**fd3& std**	14.29	2.86	**0.00**	20.00	**0.00**	2.86	28.57	11.43	5.71	14.29	5.71	5.71

**fd3 & pca**	5.71	5.71	0.00	17.14	2.86	**0.00**	22.86	**0.00**	**0.00**	11.43	5.71	8.57

**nfd3 & std**	11.43	8.57	2.86	2.86	2.86	**0.00**	20.00	8.57	8.57	5.71	2.86	2.86

**nfd3 & pca**	11.43	8.57	0.00	5.71	5.71	5.71	14.29	8.57	5.71	8.57	2.86	**0.00**

These datasets were obtained using different pre-processing and dimension reduction methods.

(For the abbreviations used in the table, see Table 2.)

**Table 4:** Classification error rates (%) at different concentrations using the Quadratic DA method.

Dataset (pre-processing & dimension reduction)	Concentration				Average error across different concentrations
		
	12 × 10^8^ cfu/ml	12 × 10^5^ cfu/ml	12 × 10^3^ cfu/ml	12 × 10^1^ cfu/ml	
**d1 & std**	0.00	0.00	2.86	2.86	**1.43**

**d1 & pca**	0.00	2.86	0.00	5.71	2.14

**fd1 & std**	2.86	5.71	2.86	0.00	2.86

**fd1 & pca**	0.00	0.00	8.57	0.00	2.14

**nfd1 & std**	0.00	0.00	5.71	5.71	2.86

**nfd1 & pca**	0.00	0.00	2.86	2.86	**1.43**

**Average for baseline value-1 (**${\text{R}}_{i}^{\text{o}}={\text{R}}_{i}^{\min}$**)**					**2.14**

**d2 & std**	2.86	11.43	8.57	11.43	8.57

**d2 & pca**	0.00	11.43	8.57	22.86	10.72

**fd2 & std**	0.00	0.00	17.14	14.29	7.86

**fd2 & pca**	2.86	2.86	2.86	2.86	2.86

**nfd2 & std**	5.71	2.86	2.86	2.86	3.57

**nfd2 & pca**	0.00	0.00	5.71	2.86	2.14

**Average for baseline value-2 (**${\text{R}}_{i}^{\text{o}}={\text{R}}_{i}^{\text{first}}$**)**					**5.95**

**d3 & std**	0.00	2.86	2.86	5.71	2.86

**d3 & pca**	0.00	0.00	2.86	11.43	3.57

**fd3& std**	0.00	2.86	5.71	5.71	3.57

**fd3 & pca**	0.00	0.00	0.00	8.57	2.14

**nfd3 & std**	2.86	0.00	8.57	2.86	3.57

**nfd3 & pca**	0.00	5.71	5.71	0.00	2.86

**Average for baseline value-3 (**${\text{R}}_{i}^{\text{o}}={\text{R}}_{i}^{\text{bp}}$**)**					**3.10**

**Average error** **across same** **concentration**	**0.95**	**2.70**	**5.24**	**6.03**	

We can deduce the following results from Table 4:

• Classification performance decreases as the concentration decreases.

• Among different baseline values used while pre-processing the sensor responses, baseline-value-1 (${\text{R}}_{i}^{\text{o}}={\text{R}}_{i}^{\min}$), where the minimum values of sensor readings in the sample were accepted as the baseline, results in better classification performance.

Corresponding to ${\text{R}}_{i}^{\text{o}}={\text{R}}_{i}^{\min}$ baseline value, we note that d1 & std (i.e., difference model with 'standard deviation based' dimension reduction) or nfd1 & pca (i.e., normalized fractional difference model with 'PCA based' dimension reduction) combination yields the top overall performance across different concentrations which averages to 1.43%. For this combination, classification error rate is only 2.86%, even for the lowest concentration of 12 × 10^1 ^cfu/ml.

## 4. Discussion and Conclusion

In this study, we aimed at classification of microorganism strains that cause root canal infections using the e-nose technology. To this end, we have cultivated the microorganisms of interest at laboratory conditions at different concentrations and obtained odor data samples. Then, we have designed DA classifiers and observed their classification performance under different conditions, in order to assess the influence of baseline values, sensor response models, and dimension reduction methods.

Our first finding is that the quadratic type DA outperforms the other two DA varieties, namely linear and Mahalanobis. This is somewhat expected, as the quadratic method lets different classes have different covariance matrices, it has more modeling capability. We still wanted to check the performance of simpler methods in order to understand the complexity of the classification problem that we are dealing with. After deciding on the quadratic DA method as the method of choice, we have looked into the influence of different factors on the classification performance. Our second finding is that as the concentration increases the classification performance improves. This is also expected, as the sensor responses will have higher amplitudes at higher microorganism suspension concentrations.

In the user's manual of the Cyranose 320 e-nose instrument, there is no specific explanation regarding the value that should be taken as the baseline value. Therefore, we have also wanted to look into the effect of using different alternatives for the baseline value (${\text{R}}_{i}^{\text{o}}$) while computing the sensor responses using formulas 1, 2, or 3. Our results revealed that the best baseline value is baseline-value-1 (${\text{R}}_{i}^{\text{o}}={\text{R}}_{i}^{\min}$), in terms of achieving a better classification performance using quadratic DA. In fact, as we observe in Tables 3 and 4, the choice of baseline value significantly affects the classification performance. For the choice of 'baseline value-1', we note that d1 & std (i.e., difference model with standard-deviation-based dimension reduction) or nfd1 & pca (i.e., normalized fractional difference model with PCA-based dimension reduction) combinations offer better classification performance, compared to other pre-processing and dimension reduction method combinations.

We have mainly concentrated on the training classification error in this study, as we wanted to find out optimal pre-processing and dimension choices for efficient use of our approach in future. Although our results indicate that there many cases for which we have obtained zero classification error rates, this does not mean that we are not going to make any wrong decisions for new test cases that were not included in the training set. Indeed, the issue of assessment of error rate of a classifier deserves much consideration [26]. During classifier design, using the information extracted from the training samples, underlying parameters of the classifier are adjusted and the prediction accuracy is monitored by testing the classifier back on the training set and noting the resultant training (or resubstitution) error. This type of assessment of classifier performance, based on training error, is instrumental during the design phase; however, it may not be an accurate indicator of the final or overall performance of the classifier. As we are interested in employing our classifier in predicting category of new or unseen samples, we also need to evaluate the generalizability performance of the classifier. If there are plenty of training samples available, one can partition the overall training set into two sets and use one for training and the other one for testing. Due to the limited number of samples we had, we did not want to reserve a portion of our dataset for testing purposes. Further, if we design the classifier based on a small training set, generalizability performance of the classifier may deteriorate significantly [27]. Instead, we have opted for 5-fold Cross Validation (CV) technique, where overall set of training samples is randomly divided into 5 approximately equal size and balanced (i.e., the distribution of samples into different classes is roughly similar) subsets. Then, each time, one of these subsets is excluded from the overall training set and used as a test set for the classifier that is trained based on the rest of the samples and the resultant test error rates are averaged to obtain the 5-fold CV error rate. Compared to training error, estimation of 5-fold CV classification error is more costly computationally. Therefore, we have carried out 5-fold CV classification error estimation for the exemplary case of 12 × 10^8 ^cfu/ml concentration and obtained a CV error rate of less than % 25, for the optimal pre-processing and dimension reduction combination that we have identified before. We envision that even a classification of accuracy of about 75% will be very appealing for the clinicians who tend to resort to empiric decision making in treatment of root canal infections, due to current limitations in the practice.

As for the issue of the existence of multiple microorganism species in the medium, we note that the decision made by our approach will reflect the major or dominant microorganism species in the medium. However, we can use the raw discriminant values as the probability of the sample under question belonging to each of the individual classes, instead of taking the maximum value of the discriminant function as the 'class decision.' This way, the clinician may see the probability of existence of each microorganism species in the medium and decide about the course of treatment accordingly. If we include the class prior probabilities, i.e., the prevalence of each microorganism species in the root canal, in computation of the discriminant function, classification performance of our approach may further improve. We are planning to look into this issue in a future clinical study.

In conclusion, we have demonstrated that the e-nose technology is a promising and convenient alternative for classifying microorganisms that cause root canal infections. With our comprehensive approach, we have also determined optimal settings in terms of which classification, pre-processing and dimension reduction methods to be used, to obtain higher classification performance using this technology. This comprehensive study is done only once to fine tune to parameters of this approach. We will use these pre-determined optimal settings or parameters in our future studies involving classification of real clinical cases. In consultation with the clinicians in our team, we will develop some suitable settings to collect odor data in a practical and efficient manner.

## Competing interests

The authors declare that they have no competing interests.

## Authors' contributions

BHA carried out data acquisition, data pre-processing and classification processes. MHA designed the study and prepared manuscript. YK and ÖE provided microorganism strains and participated in the design of the study. EK and HÖ performed preparing microorganism cultures. SK participated in the design of the study and coordination. All authors read and approved the final manuscript.
